# The complete mitochondrial genome of the Asian river pipefish *Doryichthys boaja* (Actinopterygii; Syngnathiformes; Syngnathidae) obtained using next-generation sequencing

**DOI:** 10.1080/23802359.2018.1491338

**Published:** 2018-07-11

**Authors:** Alireza Asem, Pei-Zheng Wang, Weidong Li

**Affiliations:** College of Life Sciences and Ecology, Hainan Tropical Ocean University, Sanya, China

**Keywords:** Mitogenome, Asian river pipefish, *Doryichthys boaja*, next generation sequencing

## Abstract

The complete mitochondrial genome of the Asian river pipefish *Doryichthys boaja* was sequenced. The mitochondrial genome is a circular molecule of 16,439 bp in length, containing 37 mitochondrial genes and a control region. The base composition is 31.03% A, 24.22% C, 14.44% G, and 30.32% T, with an A + T content of 61.35%. All PCGs were initiated by ATG start codon expecting *COX1* by GTG. A phylogenetic tree showed that *Doryichthys boaja* clustered with *Microphis brachyurus*.

The Asian river pipefish *Doryichthys boaja* is a freshwater fish with widely distributed in the streams, rivers, and brackish waters of Southeast Asia (Kottelat 2000; Pollom 2017). Only limited mitochondrial sequences have been published for Syngnathidae family and very few studies have been considered the phylogenetic status of this family. Here, we reported the complete mitochondrial genome of *D. boaja* (GenBank accession no. MH237607) to compare its phylogenetic platform with other members of Syngnathidae.

An adult of *D. boaja* was collected from Qionghai City (Hainan province, China) and stored in Hainan Tropical Ocean University Museum of Zoology (NO.0001-Db). The genomic DNA was extracted from dorsal-lateral muscles (30 mg) using Rapid Animal Genomic DNA Isolation Kit (Sangon Biotech Co., Ltd., Shanghai, CN; NO. B518221). A genomic library was established followed by next-generation sequencing. Quality check for sequencing data was done by FastQC (Andrews 2010) and the fragments sequences were assembled and mapped using Spades v3.9.0 (Bankevich et al. 2012).

The complete mitogenome of *D. boaja* was 16,439 bp in length, with 22 transfer RNAs (tRNAs), 2 ribosomal RNAs (rRNAs), 13 protein-coding genes (PCGs) and a control region (CR). There was a strong A + T bias (61.35%). Seven tRNAs (*tRNA-Gln*, *tRNA-Ala*, *tRNA-Asn*, *tRNA-Cys*, *tRNA-Tyr*, *tRNA-Ser*, and *tRNA-Glu*) and just *ND6* protein-coding gene were encoded on the light strand and others were encoded on the heavy strand. All PCGs began with common ATG start codon, while *COX1* was encoded with GTG. Stop codons included TAA (*ND1*, *ATP8*, *ATP6*, *COX3*, *ND4L*, *ND5*, and *ND6*), TAG (*ND2*), non-complete codons TA (*COX1*) and T (*COX2*, *ND3*, *ND4*, and *Cytb*). The *12S ribosomal RNA* and *16S ribosomal RNA* were encoded from 69 to 1011 (943 bp) and 1083 to 2754 (1672 bp), respectively, with 12S having a rather lower A + T content (57.48% vs. 60.89%). These were located between the *tRNA-Phe* and *tRNA-Leu*, and were separated by the *tRNA-Val*. The longest gap and overlapping were determined between *tRNA-Asn*/*tRNA-Cys* (35 bp) and *ATP8*/*ATP6* (10 bp), respectively.

The phylogenetic status of *D. boaja* among Syngnathidae family was determined from a concatenated dataset including the 2 rRNAs and 13 PCGs using the software MEGA 7.0.26 v. (Kumar et al. 2016) with 1000 bootstrap replicates and GTR model (Figure 1). According to the result of phylogenetic tree, the studied sample showed a close evolutionary relationship with *Microphis brachyurus* in comparison with others. It needs to sequence the mitogenomes of other members of Syngnathidae to determine the phylogenetic position among them.

**Figure 1.**
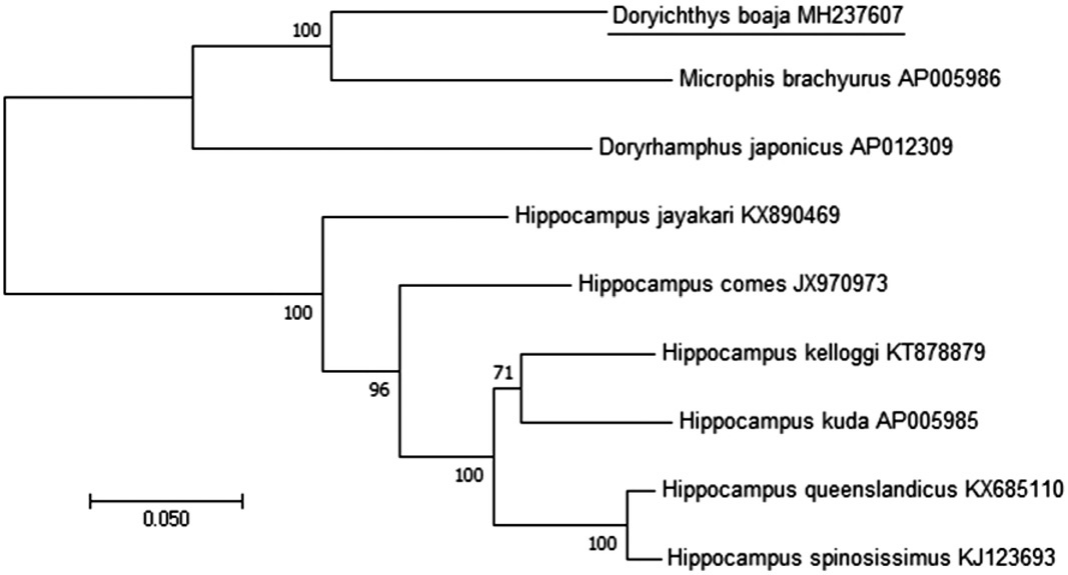
Phylogenetic tree showing the relationship among *D. boaja* and 9 other species of Syngnathidae based on maximum-likelihood (ML) approach. Numbers behind each node denote the bootstrap support values. The GenBank accession numbers are indicated on the right side of species names.
